# Virtual Fencing Is Comparable to Electric Tape Fencing for Cattle Behavior and Welfare

**DOI:** 10.3389/fvets.2019.00445

**Published:** 2019-12-11

**Authors:** Dana L. M. Campbell, Jim M. Lea, Hamideh Keshavarzi, Caroline Lee

**Affiliations:** Agriculture and Food, Commonwealth Scientific and Industrial Research Organisation (CSIRO), Armidale, NSW, Australia

**Keywords:** stress, cortisol, associative learning, time budget, body weight

## Abstract

Virtual fencing technology restricts animal movement via communicated signals without physical boundaries. Specifically, the eShepherd™ automated virtual fencing system operates via GPS technology and provides stimuli via a neckband device. An audio warning tone is emitted at the virtual boundary which is followed by an electrical pulse if the animal continues moving forward. Animal welfare is a priority consideration for the commercial implementation of virtual fencing systems. The current study assessed the effects of a virtual fence, in comparison to an electric tape fence, to contain eight groups of eight 12–14 month old steers within a 6-ha area across eight separate paddocks for 4 weeks following 1 week acclimation to the paddocks. Cattle were assessed across two cohorts (four groups/cohort) from January until March 2019 in Australia. Body weight and fecal samples from each animal were taken weekly. Fecal samples were processed for fecal cortisol metabolite (FCM) concentrations. IceQube R®'s fitted to the leg measured individual lying and standing time and the virtual fencing neckbands recorded GPS location and all administered audio and electrical stimuli. Cattle were maintained within their allocated area by both fence types across the 4-week period and those with the virtual fences were responding correctly to the audio cue with an average of 71.51 ± 2.26% of all cues across all animals being audio only. There was individual variation in rate of learning. The electric tape groups in cohort 1 showed a greater increase in body weight over 4 weeks than the virtual fence groups (*P* < 0.001) but this difference was not confirmed in cohort 2. The fence type statistically influenced the total daily lying time (*P* = 0.02) with less lying in cattle from the virtual fence groups but this difference equated to an average of <20 min per day. There were no differences between fence types in FCM concentrations (*P* = 0.39) and the concentrations decreased across time for all cattle (*P* < 0.001). These results indicate that virtual fencing technology effectively contains animals in a prescribed area across 4 weeks without substantial behavioral and welfare impacts on the cattle.

## Introduction

Virtual fencing technology has the potential to revolutionize management of the livestock industries. The presence of a virtual fence is communicated to the animals via signals rather than through the presence of a physical barrier which can increase the flexibility of fencing options. This may result in reduced labor, improved herd management, and protection of environmentally-sensitive areas. Multiple experimental prototype models have proven the application potential of virtual fencing but few systems have been developed for commercial use (1). The eShepherd™ virtual fencing system uses licensed IP developed by the Commonwealth Scientific and Industrial Research Organization (CSIRO) (2, 3) and is being commercialized for use with cattle by Agersens (Melbourne, VIC, Australia). The eShepherd™ system operates using GPS technology, sending signals via radio link to individual cattle wearing a device on a neckband. The location of a virtual fence is communicated to the animal using an audio tone when the animal approaches a set virtual boundary. This audio tone is followed by an electrical pulse if the animal continues moving toward the virtual boundary. The animals are trained on the principle of associative learning where the correct response to the audio tone (stopping or turning away) will prevent administration of the electrical pulse (4, 5).

Previous trials conducted with the pre-commercial prototype of the eShepherd™ system have demonstrated that animals can be contained by the virtual fence and that they rapidly learn the association between the stimuli and start avoiding the boundary based on the audio cue alone, thus avoiding receiving electrical stimuli (6–8). Trials have also demonstrated that when a virtual fence is periodically shifted (every few days) or deactivated, the cattle will move into previously excluded areas within a few hours following the absence of the signals that they previously received (6, 8). Individual animals vary greatly in both their rate of associative learning and how frequently they interact with the virtual boundary with potential effects of social facilitation when the animals are exposed to the virtual fence in groups vs. individually (6–8).

Animal welfare is a priority consideration for the commercial implementation of virtual fencing systems. For this technology to be successful at the farm level, and widely adopted with social license approval from the general public, it must be animal-friendly and adhere to high welfare standards. Demonstration of acceptable animal welfare standards would include ensuring that all animals are able to learn the association between the cues and respond to the audio cue alone so that their environment is controllable and predictable (9). Cattle also exhibit typical behavioral patterns (time budgets) while at pasture that consist of mostly grazing, ruminating, and resting although exact time spent engaged in each behavior varies significantly between study groups (10, 11). While behavioral patterns can vary due to factors such as pasture availability, social and climatic conditions, deviations from “normal” patterns, such as reduced lying time, can indicate discomfort and stress which can lead to physiological health consequences (12–14). Physiological measures such as increased fecal cortisol metabolite (FCM) concentrations can also be indicative of anxiety in cattle (15), negative environmental impacts (16), or a lack of FCM differences between treatment groups can confirm minimal impacts of different experiences (17, 18).

To date the research studies with the eShepherd™ pre-commercial system on beef cattle have included small samples of animals [maximum 12 for a single trial, (8)] and across short periods of time [maximum 16 days in a single trial, (6)]. Campbell and colleagues (6) reported some changes across time in lying bouts when a moving virtual fence was used with a small group of beef cattle but there was no control group comparison in this study. Controlled research on the aversiveness of the electric pulse itself showed that animals receiving a mild pulse while in a crush had similar behavioral and physiological stress responses to being head-restrained and returned to normal behavior within 10 min of treatment (19). Physical fencing systems that use electrical stimuli as a deterrent such as electric wires or electrical tape are applied frequently across cattle and other livestock industries. These operate under similar learning principles to the virtual fence, but the conditioned stimulus to avoid (the fence) is visual rather than audio. Early trials demonstrated cattle in groups learn about wire electric fences in a similar manner to virtual fences where most interactions occur on the first day, each animal only has a few interactions in total, and some individuals stay within the prescribed area without touching the fence, presumably socially learning to avoid the barrier (20–22). A comparative assessment between virtual fencing (audio cue signal) relative to physical electric fences (visual signal) is informative for determining the impacts of this new technology on cattle behavior and welfare.

The objectives of the current study were to use the eShepherd™ system to virtually fence beef cattle for a period of 4 weeks in comparison to electrical tape fencing. Measurements included GPS paddock-use patterns, behavioral time budgets, FCM concentrations, body weight, and associative learning rates of those groups with virtual fences. It was predicted that all animals would learn to respond to the audio cue alone but with individual variation in learning rate and that there would be minimal differences between the virtually-fenced and electrically-fenced groups in the measures of behavior and welfare.

## Materials and Methods

### Ethical Statement

The experiment was approved by the CSIRO FD McMaster Laboratory Chiswick Animal Ethics Committee (ARA18/25) prior to the start of the experimental period.

### eShepherd™ Neckbands

The virtual fencing pre-commercial prototype (eShepherd™, Agersens, Melbourne, VIC) system was used in these trials and has been described previously (8). The neckband that the cattle wore consisted of a strap and hanging counterweight (total weight ~1.4 kg) and a unit (~725 g and 17 cm L × 12 cm W × 13 cm H), positioned on the top of each animal's neck. Using GPS technology, the unit monitored the animal's movement to provide a real-time measure of the animal's position, heading and speed. A virtual fence boundary (separating inclusion vs. exclusion zones), was specified using GPS coordinates, and transmitted to the unit using a radio frequency link. As an animal approached the virtual fence boundary the unit emitted a distinctive but non-aversive audio tone within the animal's hearing range. If the animal stood still or turned away, no electrical pulse was applied. If the animal continued to move through the virtual fence boundary into the exclusion zone, the unit delivered a short, sharp electrical pulse sequence in the kilovolt range (values are commercial in confidence). The intensity of the pulse stimulus delivered by the neckband was lower in energy than an electric fence. Additionally, because the neckband was worn by the animal, the pulse was delivered via a different mechanism than an electric fence. Hence no direct comparison can be made of pulse intensity between a neckband and a standard electric fence. This sequence of an audio cue followed by the electrical pulse was repeated if the animal walked through the fence line and continued into the exclusion zone. No stimuli were applied if the animal turned around to re-enter the inclusion zone. If movement occurred above or below a specified velocity (values are commercial in confidence), stimuli were not applied. If an individual animal received a specified number of stimuli within a specified time frame, the device entered standby mode and stimuli were not applied for a specified time frame (values are commercial in confidence). The neckband algorithm also included a grazing function. The natural behavioral pattern of grazing can mimic the correct response by the animal to the neckband cues of movement forward and stopping at an audio cue. Therefore, if an animal gradually encroached on the exclusion zone by grazing, after three consecutive audio cues while slowly moving forward paired with stopping, an electrical pulse was applied. A base station was set up adjacent to the trial paddocks that communicated with the neckbands and animal activity was able to be monitored in real time through an online user-interface.

### Animals and Experimental Protocol

**Figure 1:**
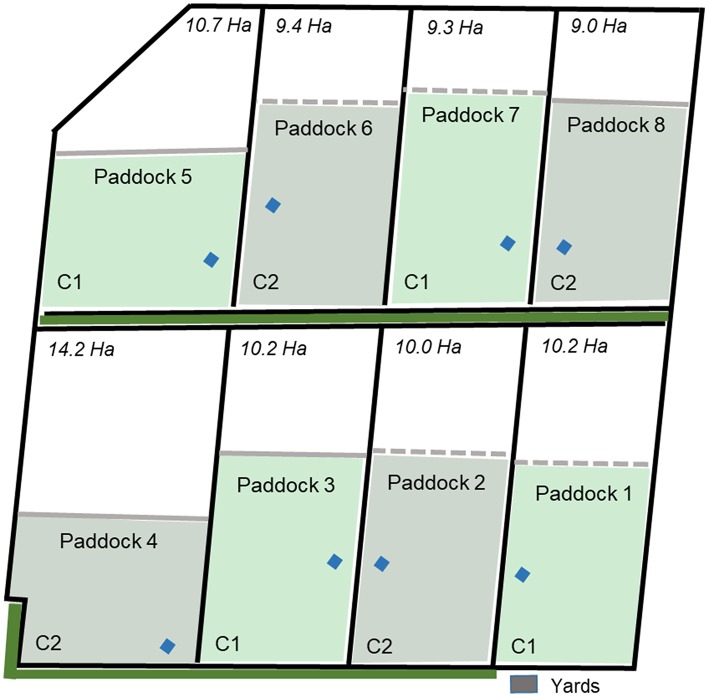
The numbered experimental paddocks showing total paddock size (hectares), the placement of electric tape fences (solid line) or virtual fences (dashed line), the assigned cohort (C1, C2), location of the yards, water points (blue diamonds), and tree line (solid green line) outside the paddock physical fences. Each fenced inclusion zone was 6 ha in size.

A total of sixty-four 12–14 month old Angus steers were used in the trial divided into eight groups of eight animals each. The commercially-reared animals came from an established Angus herd of 1,500 cows using top industry bloodlines. They were yard-weaned for 1 week and had no prior exposure to electric tape or virtual fences. Animals were brought onto the research site in Armidale in December 2018. No formal temperament tests were conducted but experienced cattle handlers on site deemed the animals to require handling and personnel exposure before they were ready to be fitted with neckbands in January and handled regularly across the trial period. Animals were tested in two separate, sequential cohorts with 4 groups tested per cohort. In January 2019, the first 32 animals were weighed in the crush (Tru-Test XR3000, Tru-Test, Banyo, QLD, Australia), fitted with eShepherd™ neckbands, an IceQube R® (IceRobotics Ltd, Edinburgh, Scotland, UK) on the front leg, and painted with a unique number on each flank using livestock tail paint (Leader Products Pty Ltd., Craigieburn VIC, Australia). The heavier animals within the total group were selected for testing in the first cohort to ensure the most optimal neckband fit. As the animals were not accustomed to handling, they had adverse responses to being in the yards and race. Accordingly, the time taken to initially weigh the animals was kept to a minimum to ensure animal and personnel safety. While animal distribution into treatment groups was randomized as best as logistically possible, the initial difficulties in handling resulted in the distributions by weight being not as precise as desired. Each group was then placed into four different paddocks ~10 ha in size each, located at the Commonwealth Scientific and Industrial Research Organization (CSIRO) Chiswick site in Armidale, NSW (Figure 1). Cohort 1 was placed into paddocks 1, 3, 5, 7 (Figure 1), with cohort 2 placed into the remaining four paddocks immediately following the removal of the first groups. An empty paddock was always between different groups to minimize visual contact. The animals were given 9 days free access to the entire paddock area to acclimate to the new environment with grazing pasture and water freely available. There was a single tree line along one side of 7 of the 8 paddocks (Figure 1). This was outside the physical fences but trees overhung the paddocks. The neckbands continually recorded GPS location data throughout the day at different sampling intervals (seconds or minutes) dependent on the animal's distance from the virtual fence. The IceQubes® recorded information on lying time, standing time, and lying bouts across 24 h intervals (the devices also recorded the number of steps but these data were not used in the analyses for this study).

**Figure 2:**
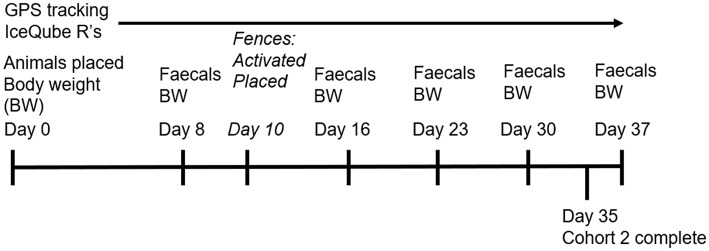
A depiction of the experimental data collection timeline for cattle tested within two cohorts. NB: Cohort 2 finished 2 days earlier than cohort 1 due to neckband malfunction.

On day 8 while moving animals to the yards for weighing (Figure 2), fresh fecal samples were collected from each individually-identified animal as they defecated and immediately placed into a portable freezer (Engel fridge/freezer 40 L, model MT45FP, Sawafuji, Ota city, Japan). On the morning of day 10, single virtual fence lines were set across the width of two test paddocks, and solar-charged electric tape (6,000–7,000 kV 12 V Speedrite, 0.25 J Model) was placed across the width of the remaining two test paddocks reducing each available paddock area to 6 ha (Figure 1). Personnel were present within the adjacent empty paddock to observe the first interactions of the groups with the electric tape. The neckbands with activated virtual fences recorded the number of audio and electrical stimuli received by each individual animal every time they interacted with the virtual fence.

The fences remained in place for a period of 27 days (day 10 to day 37). Animals were walked to the yards for weighing again on days 16, 23, 30, and 37 and fecal samples were also collected from each animal on those days (Figure 2). On day 37 the trial for the first cohort concluded (total 36 days in test paddocks). The second cohort of animals followed the same procedure and the four groups were placed into four different paddocks (Figure 1) in February 2019 with the fences activated/placed on day 10. Weighing occurred on days 8, 16, 23, 30, and 37 with fecal samples also collected on those days except for the final fecal sample which was collected on day 35 (Figure 2). The behavioral data collection also ceased on day 35 for this cohort due to a neckband malfunction which resulted in early trial termination. Across both cohorts, if a neckband unexpectedly came off the animals in the electric tape group then the neckband was not placed back on until the next weighing day to minimize disturbance. However, it was necessary to replace the neckband on the same day for the virtual fence groups to minimize the risk of an animal without a virtual fence drawing fenced animals into the exclusion zone. For replacement, the entire group was moved down to the yards. For balance, the remaining groups were moved around their paddocks for an equal period of time to account for potential effects on cortisol. This happened on a total of three occasions for cohort 1 and two occasions for cohort 2. The fences for the two electric tape groups in cohort 2 were shifted to increase paddock size by 1 ha on day 30 of the trial to expose some more pasture for animals in these paddocks for the remainder of the trial as they had shown a decrease in body weight. The virtual fence was not moved as the animals in these paddocks were not exhibiting the same decrease in body weight and any increased grazing near the shifted line may have subsequently impacted the degree of interaction with the virtual fence at the later trial stages.

The average minimum, overall, and maximum temperatures across the trial period for cohort 1 were: mean ± SEM min: 14.56 ± 0.45°C, avg: 22.17 ± 0.35°C, max: 30.93 ± 0.50°C and mean ± SEM min: 12.67 ± 0.35°C, avg: 18.83 ± 0.44°C, max: 26.64 ± 0.59°C for cohort 2 based on weather data collected directly at the Chiswick site.

### Fecal Sample Processing

Fecal samples were collected per individual animal within the same 2–3 h period in the morning at the end of each trial week (acclimation, and 1, 2, 3, 4 weeks post fence activation/placement). Samples were later oven-dried at 60 degrees centigrade for 48 h and ground using a Retsch Ultra Centrifugal Mill (ZM 200) with a 2 mm sieve. The individual samples were then processed at the University of Western Australia. Around ~100 mg of dried sample were reconstituted in 300 μl of DD water followed by vortex for 5 min. This was added to 2,700 Ml of 100% ethanol, vortexed for 10 min, then spun at 2,000 G for 10 min; the supernatant was decanted in glass tubes. Pellets were extracted again with 3 ml of 100% ethanol, spun at 2,000 G for 10 min and the supernatant added to the previous extract. The extracts were dried under airflow for 5–6 h and then were reconstituted in 500 μl of Phosphate buffer Saline (pH 7.4), vortexed for 10 min and spun at 1,000 G for 2 min. Extraction efficiency was 86 ± 3%. The limit of detection was 2.5 ng/ml and the mean inter-assay coefficients of variation were 8.1 and 6.6%. Concentrations of fecal cortisol metabolites (FCM) in the extract were measured in duplicate using the MP Biomedical I125 RIA cortisol Kit (# 07-221106) (MP Biomedicals Australia, Seven Hills, NSW). A total of 320 individual samples were analyzed. Results are expressed as nanograms of FCM per gram of dry feces.

### Data and Statistical Analyses

The neckbands did not record data on some days for some animals, or for some portions of the day for some animals due to technical malfunction. These neckbands still delivered the signals to the animals but there were errors with internal data storage. Of the total 2,176 days of recording for 64 animals (35 and 33 days for cohort 1, and 2, respectively), there were 48 full missing days across 27 animals and 44 partial missing days across 19 animals. All GPS data recorded by the neckbands were compiled in SQL Server software (23). Spurious points far outside the paddocks indicative of GPS drift were removed but some GPS error around the paddock boundaries still remained as per standard GPS accuracy. All available locational data (including partial days) were plotted in the R statistical package (24) per week for each groups of animals. GPS data were recorded every second when the animals were within a specified distance (value commercial in confidence) from the virtual fence line and/or walking/grazing.

The total number of received audio cues and pulses per animal per day was also calculated for the virtual fence groups. The number of received signals for each animal was considered to be null if the neckband had worked for <16 h of the total 24 on that day. In total, due to neckband malfunction, there were 51 days missing (7 full, and 44 partial missing days) from 816 study days of records for the virtual fence groups (16 neckbands × 27 study days + 16 neckbands × 24 study days). The average number of received audio and electrical pulse cues per week was calculated for all animals in each paddock, including the percentage of audio-only cues received. The average number of audio/pulse combinations experienced before individual animals first responded appropriately to the audio cue alone was calculated. Any responses to the audio cue alone before receiving an electrical pulse were disregarded as these were presumably as a result of social effects rather than associative learning.

Individual body weight data were compiled for each weighing week per group of animals tested (*n* = 384: 8 groups × 8 individuals × 6 weighing periods). The change in body weight was calculated from placement until the end of the trial (week 5) for cohort 1, and from placement until week 4 only for cohort 2 due to the shifting of the tape fence to expose more pasture (mean weekly values are presented in the tables). Behavioral data from the IceQube R's® were compiled into mean daily standing time (minutes), lying time (minutes), and lying bouts per animal per week with the first day of all groups and the last day of groups in cohort 1 excluded as they were not a full 24 h of recording (*n* = 315: 8 groups × 8 animals × 5 weeks − 5). One IceQube R® did not function for one animal in a virtual fencing group of cohort 1. The lying bout count data were square-root transformed. The concentrations of cortisol metabolites in dry feces (ng/g) were compiled per individual animal per sample week (*n* = 320: 64 animals × 5 sample periods). General Linear Mixed Models (GLMM) were applied to the behavioral and FCM datasets to compare the fixed effects of cohort, fence type, trial week, and their interaction with animal ID nested within group and fence type as a random effect. Restricted maximum likelihood estimation methods were applied. Non-significant interactions were removed from the final models. For the change in body weight data, cohorts were analyzed separately with the fixed effect of fence type and random effect of animal ID nested within group and fence type. Where significant differences were present, *post-hoc* Student's *t*-tests were applied to the least squares means with Bonferroni correction applied to the alpha level for multiple comparisons. All analyses were conducted in JMP 14.0 (SAS Institute Inc., Cary, NC, USA) with α set at 0.05.

## Results

**Figure 3:**
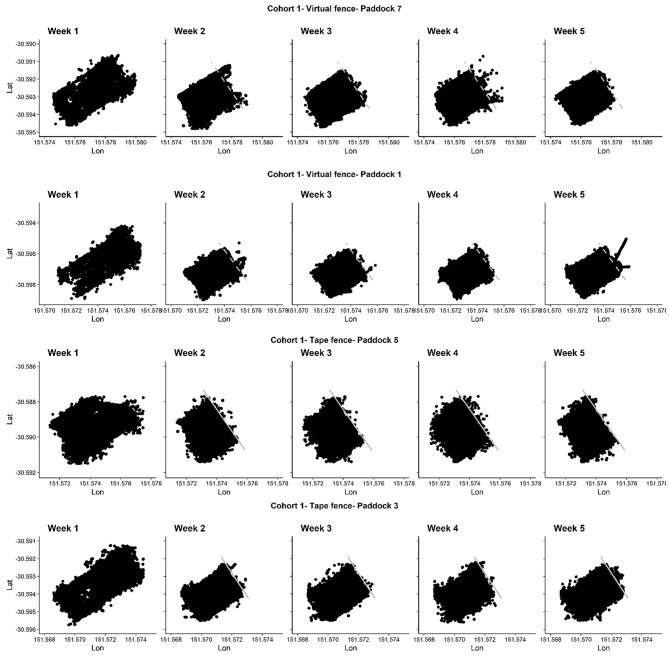
The GPS locations (latitude and longitude) of four cattle groups in cohort 1 across each week of the trial. An electric tape fence (solid lines) or a virtual fence (dashed lines) was placed/activated at the beginning of week 2. The fence type and paddock number (as related to Figure 1) is indicated. On week 2 in paddocks 1 and 7 cattle did cross over the virtual lines as they were learning the cues. Dots are GPS locations of individual cattle that were recorded every second when the animals were within a specified distance (value commercial in confidence) from the virtual fence line and/or walking/grazing. The shape of the GPS dots aligns with the shape of the paddocks as per Figure 1 indicating cattle used all available areas during week 1 and were subsequently restricted by the fence. Dots outside of the paddock boundaries indicate typical GPS error. In week 5 in paddock 3, an animal pushed under the electric tape twice due to a short in the fence.

**Figure 4:**
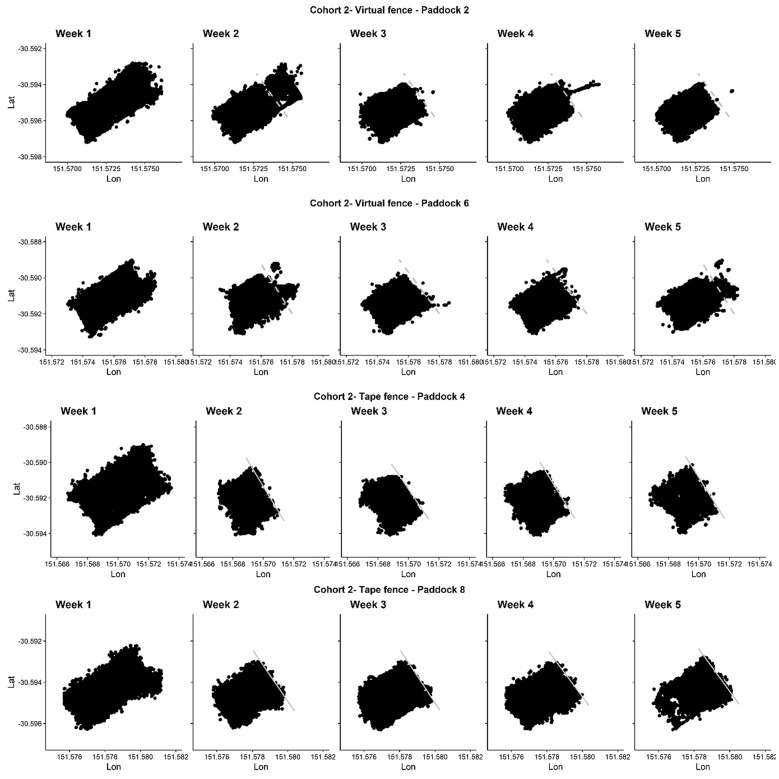
The GPS locations (latitude and longitude) of four cattle groups in cohort 2 across each week of the trial. An electric tape fence (solid lines) or a virtual fence (dashed lines) was placed/activated at the beginning of week 2 (NB: week 5 in cohort 2 consisted of only 3 days due to early experiment termination). The fence type and paddock number (as related to Figure 1) is indicated. On week 2 in paddocks 2 and 6 cattle did cross over the virtual lines as they were learning the cues. Dots are GPS locations of individual cattle that were recorded every second when the animals were within a specified distance (value commercial in confidence) from the virtual fence line and/or walking/grazing. The shape of the GPS dots aligns with the shape of the paddocks as per Figure 1 indicating cattle used all available areas during week 1 and were subsequently restricted by the fence. Dots outside of the paddock boundaries indicate GPS error. On week 5 in paddock 6, an extra 7 h of recording past the fence shut down is displayed (fence was turned off at 17:00, the neckbands recorded until 00:00) showing animals crossing into the previously excluded area. On week 5 in paddocks 4 and 8, the electric tape was shifted to expose more grazing area (to compensate for a decrease in body weight). The virtual fences were not moved as these animals were not exhibiting the same decrease in body weight and any increased grazing near the shifted line may have subsequently impacted the degree of interaction with the virtual fence at the later trial stages.

Cattle utilized the entire paddock areas during their acclimation week (week 1 in Figures 3, 4). Both the electric tape and virtual fences were successful in keeping animals within their prescribed areas for the majority of the time (Figures 3, 4). Animals did cross over the virtual boundary when they were first learning the signals which aligns with previous findings (weeks 2–5 in Figures 3, 4). Observations made for three of the four groups of the first exposure to the electric tape showed that the majority of animals approached the tape, either touching it to receive an electrical pulse, or placing their noses directly up to it and turning away. No extreme reactions were observed (e.g., running, circling, vocalizing), no animals broke through the tape, and observations of other herd members did not appear to deter an individual from investigating the fence themselves.

Across the 4-week fenced period there were ventures into the exclusion zone for the virtually-fenced animals, particularly across the first week (Figures 3, 4). There was one animal that pushed under the electric tape twice in week 5 (paddock 3) due to a short in the fence. Animals in the virtual fence groups spent <3% of their time in the exclusion zone.

**Figure 5:**
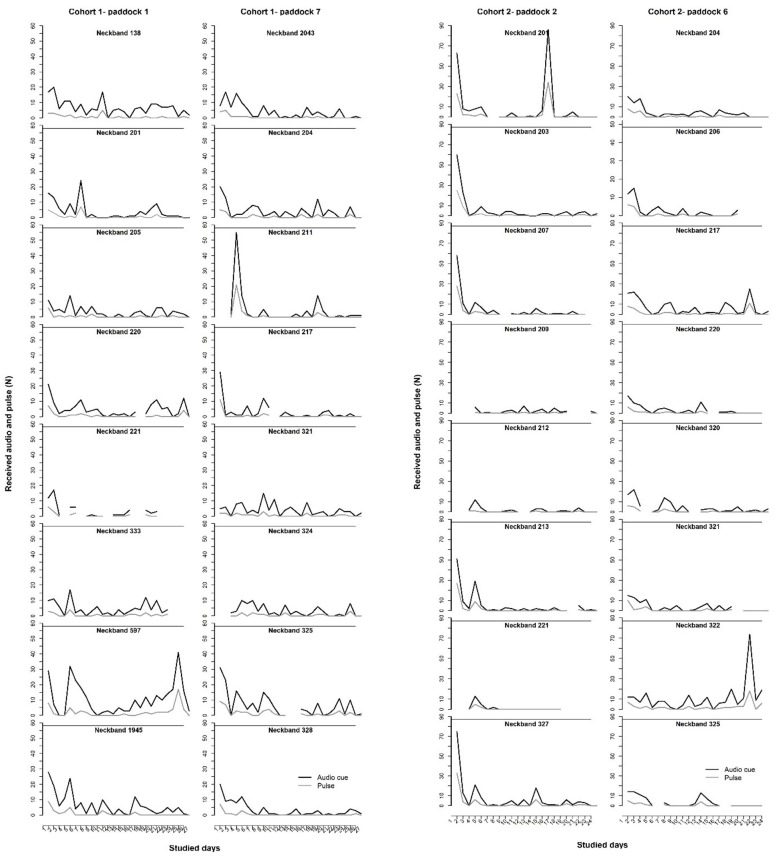
The audio cues and electrical pulses received by each individual animal across the trial duration for each of the four virtual fence groups (indicated by paddock number) across two cohorts. Missing values on specific days are where the neckbands missed recordings, this occurred frequently with neckband 221 in particular. Neckbands were still functional and delivering cues when recordings were missed. NB: different scales on the y-axes across the two cohorts and fewer recorded days for cohort 2.

**Figure 6:**
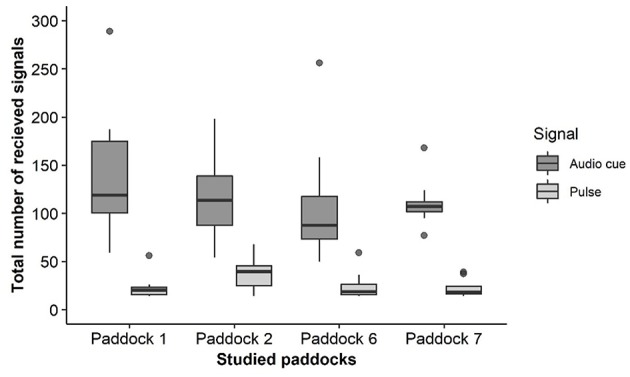
Box plots of the total number of received audio and electrical pulse signals for the four test groups across the trial duration. Paddocks 1 and 7 were part of cohort 1, and paddocks 2 and 6 were part of cohort 2.

All animals interacted with the virtual fence across the trial duration and received more audio than electrical pulse cues (mean ± SEM 71.51 ± 2.26% of all cues across all animals were audio only), but with individual variation in both learning and number of fence interactions (Figures 5, 6). On average, individuals experienced 2.5 (range 1–6) audio cue/electrical pulse combinations before they first started responded appropriately to the audio cue alone. There were fewer interactions with the fence as the trial progressed (Figure 5). The average percentage of audio-only cues received as a total of all received audio signals (paired with an electrical pulse) varied between groups with all groups showing more audio-only cues in weeks 3 and 4 than week 2, but a decrease again in week 5 for some groups (Table 1).

**Table 1:** The mean ± SEM percentage of audio-only cues that were received for each group of animals (indicated by paddock) within two cohorts across 4 weeks following virtual fence activation.

	**Cohort 1**		**Cohort 2**	
	**Paddock 1**	**Paddock 7**	**Paddock 2**	**Paddock 6**
Week 2	73.31 ± 2.10	74.38 ± 4.0	58.29 ± 4.67	49.03 ± 4.32
Week 3	80.25 ± 5.20	83.33 ± 2.91	83.44 ± 3.46^1^	81.33 ± 3.73
Week 4	88.84 ± 1.89	87.18 ± 1.63	79.13 ± 5.42	76.31 ± 4.61
Week 5	80.08 ± 6.18	72.02 ± 5.84	67.91 ± 7.88^1^	65.21 ± 9.42^2^

1, 2 *Indicates the number of animal data points that were removed for that week if the percentage of audio-only cues was zero, but the animal only received one paired cue for that week. The percentages with these animals included were 73.0 ± 10.85 (paddock 2, week 3), 59.42 ± 22.07 (paddock 2, week 5), and 40.76 ± 13.2 (paddock 6, week 5)*.

There was a significant effect of fence type on the change in body weight for cohort 1 [*F*_(1, 30)_ = 25.35, *P* < 0.0001] with the electric tape groups in cohort 1 showing a greater increase in body weight than the virtual fence groups (mean ± SEM electric tape: 48.94 ± 2.45 kg, virtual fence: 32.38 ± 2.20 kg) but the electric tape animals were of a slightly higher starting live weight (Table 2). This difference was not observed in cohort 2 [*F*_(1, 30)_ = 0.71, *P* = 0.41] (mean ± SEM electric tape: 16.94 ± 2.65 kg, virtual fence: 20.19 ± 2.80 kg). In general, cattle increased in body weight across time, but cattle in cohort 2 started to decline in body weight toward the end of the trial (Table 2). As indicated in the methods, the electric tape fence was shifted to expose more pasture following the first indication of weight drop in these groups. All cattle were moved out of the trial paddocks onto fresh pasture at the conclusion of the trial.

**Table 2:** The body weight and FCM in dry feces for cattle exposed to a virtual or electric tape fence within a 5-week period across two test cohorts^1^.

**Variable**	**Fence**	**Placement**	**Week 1**	**Week 2**	**Week 3**	**Week 4**	**Week 5**
Body weight (kg)^2^	Virtual C1	385.8	395.1	403.6	408.8	410.3	418.2
	Tape C1	395.2	419.8	422.3	438.7	435.5	444.1
	Virtual C2	387.0	399.3	403.4	405.9	407.2	398.6
	Tape C2	369.6	383.4	390.1	390.8	386.6	381.8^5^
FCM (ng/g)^3^^,^ ^4^	Virtual		25.19^a^	17.72^b^^,^ ^c^	14.84^c^^,^ ^d^	13.76^d^^,^ ^e^	10.46^e^
	Tape		23.75^a^	22.17^a^^,^ ^b^	18.87^b^^,^ ^c^	16.15^c^^,^ ^d^	10.24^e^

1 The fences were set at the beginning of week 2, fecal samples were collected at the end of each week.

2 *Values are presented as the least squares means ± 5.6 as the standard error in cohort 1 (C1) and 5.9 as the standard error in cohort 2 (C2)*.

3 *Values are presented as the least squares means ± 1.13 as the standard error of the mean*.

4a−e *Dissimilar superscript letters indicate differences between fence types across the trial weeks (P < 0.005)*.

5 *The electric tape was shifted to expose more pasture during week 5 for cohort 2*.

**Figure 7:**
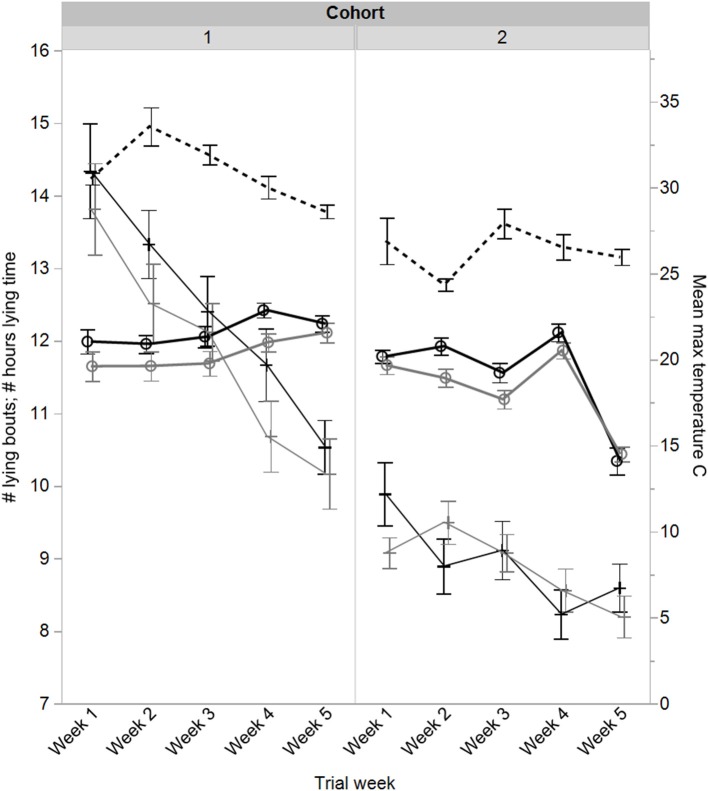
Mean ± SEM daily lying time (hours) and number of daily lying bouts of cattle in the electric tape and virtual fence groups across the weeks of the trial for two cohorts. Mean ± SEM maximum temperatures (°C) are indicated for each trial week. The fences were set at the beginning of week 2. Raw data are presented.

There was a significant difference in how lying changed across the trial weeks for cattle experiencing the two fence types [*F*_(4, 240)_ = 2.91, *P* = 0.02, Figure 7], and also across trial weeks between the two cohorts [*F*_(4, 240)_ = 64.22, *P* < 0.0001, Figure 7]. Overall, cattle from the virtual fence groups were lying less than cattle from the electric tape groups [*F*_(1, 60)_ = 5.56, *P* = 0.02, Figure 7; mean ± SEM total lying electric tape: 11.84 ± 0.07 h, virtual fence: 11.57 ± 0.08 h]. There were no interactions between cohort, trial week, and fence type (*P* = 0.53), or cohort and fence type (*P* = 0.66); these were removed from the final model. The same (opposing) pattern of results were found for standing time which was recorded by the IceQube R®'s as a corresponding opposite to any time spent lying.

There was a significant interaction between cohort, trial week, and fence type for the number of lying bouts [*F*_(4, 240)_ = 3.39, *P* = 0.01] with more lying bouts in cohort 1 and a similar reduction across time for both fence types, but different patterns between fence types across time in cohort 2 (Figure 7). There was a significant interaction between cohort and trial week [*F*_(4, 240)_ = 14.76, *P* < 0.0001], an overall effect of cohort [*F*_(1, 60)_ = 79.40, *P* < 0.0001], and trial week [*F*_(4, 240)_ = 72.0, *P* < 0.0001] but no overall difference between fence types [*F*_(1, 60)_ = 0.68, *P* = 0.42, Figure 7]. There were no significant interactions between trial week and fence type (*P* = 0.35), or cohort and fence type (*P* = 0.47); these were removed from the final model.

There was a significant interaction for the change in FCM concentrations across time between fence types [*F*_(4, 244)_ = 3.74, *P* = 0.005, Table 2] but no overall effect of fence type [*F*_(1, 61)_ = 2.97, *P* = 0.09 mean ± SEM electric tape: 18.24 ± 0.76 ng/g; virtual fence: 16.39 ± 0.76 ng/g]. FCM concentrations decreased across time [*F*_(4, 244)_ = 62.48, *P* < 0.0001] with the highest concentrations during the acclimation period (Table 2). Cohort 1 also had significantly higher overall concentrations compared with cohort 2 [*F*_(1, 61)_ = 150.67, *P* < 0.0001 mean ± SEM cohort 1: 23.87 ± 0.76 ng/g; cohort 2: 10.76 ± 0.76 ng/g]. There were no significant interactions between cohort and fence type (*P* = 0.14), or between cohort, week, and fence type (*P* = 0.43) and these were removed from the final model.

## Discussion

This trial demonstrated that Angus beef cattle could be restricted to a specified paddock area over a period of 4 weeks by both electric tape and virtual fencing. The two fence types had similar impacts on the behavior of the cattle in terms of the paddock area they used, their lying bouts and their stress physiology as assessed by fecal cortisol metabolite (FCM) concentrations. The virtual fence groups showed statistically less lying time than the electric tape groups but were still within typical cattle lying time ranges. The electric tape groups in cohort 1 showed a greater increase in body weight than the virtually-fenced groups but this pattern was not confirmed in the second cohort and may have been related to variation in paddock feed availability. All animals exposed to the virtual fencing system learned to appropriately respond to the audio cue alone across time to minimize receiving electrical pulses but with high individual variation in learning. Overall, virtual fencing technology represents an alternative fencing strategy that does not appear to adversely affect cattle behavior and welfare as assessed by measures used within this study.

All groups of animals showed utilization of all accessible areas of the paddocks when the virtual or physical electric fences were applied. This indicated they were not avoiding the locations of either type of fence line. In a previous virtual fencing study using a different system applied to strip-grazed dairy cattle, some social changes were observed compared with electrical fencing for strip grazing. Both inter-individual distances and group behavioral synchronization were lower in the virtual fence over electric fence groups using a buried wire system (25). The different type of signaling used in the buried wire system may have resulted in poor learning and elevated stress if the system does not allow the animal to be in control of its environments (9) but only limited detail was available on the precise functioning of the cues of this different system used by Koene et al. (25). In a separate study using earlier prototypes of the same system as the current trial, GPS tracking of cattle showed some indication of greater time spent close to physical fence lines and uneven paddock utilization [see Figure 2 in (6)]. This may have resulted from social changes relative to the virtual fence, but there were no control comparisons in that study (6). No distinct movement pattern differences between fence types were confirmed by the current study. This suggests the cattle were responding to the visual cue of the electric tape, and the audio cue of the virtual fencing system with no specific area avoidance. However, further study could assess inter-individual proximity to determine any social structure changes relative to fence types.

Utilization of all areas of the paddock could also be related to the pasture availability. The paddocks the cattle went into were determined to have sufficient pasture (visual assessment) to maintain 8 cattle for the trial duration, but there was not an overabundance of feed. The Armidale area had been experiencing a drought and although the paddocks were kept empty for ~6 weeks prior to the cattle placement to allow pasture regrowth, the lack of rain limited the extent of grass growth. Weekly body weight assessments confirmed the cattle were gaining weight, but this gain did reverse in the second cohort leading to the need to move the electric tape to expose more pasture area in the final week. The virtual fence was not moved as these animals did not show the same weight loss and it was decided that a shift in the virtual line may have affected the number of interactions with the fence in the final week which was outside the objectives of the study. The paddocks for cohort 2 had been empty for 10 weeks by the time the animals were placed, but the weather across this period was hot and dry affecting growth; no formal pasture assessments were made. The paddock usage patterns may potentially have been different if the cattle had been able to meet their daily feed needs within limited areas. The changes across the trial in daily lying and standing time, as well as the differences between the electric tape and virtual fence groups may have also been related to pasture availability. Particularly for cohort 2, where there was a sharp increase in standing time for both fence types in the final week. This may have resulted from increased grazing time in response to the fence shift in the electric tape groups, and higher grazing pressure in the virtual fence groups. Formal pasture assessment across study weeks would confirm any relationship between time budgets and feed availability.

The virtually-fenced animals showed statistically significantly reduced lying time compared with the electric tape groups, but biologically this equated to an average of <20 min difference per day. Cattle (beef and dairy) can vary individually in total lying time within the same farm (14) as well as between different study groups [e.g., (26, 27)] but require around 12 h of lying time (28). “Normal” beef cattle behavior while at pasture should include the majority of time spent grazing, ruminating, and resting (10). Grazing and ruminating were not measured in this study but lying time of cattle exposed to both fence types exceeded 11 h. Previous work with dairy cattle using a different type of virtual fencing system also showed reduced lying time in the virtually-fenced compared with electrically-fenced cattle, but the differences were greater than those seen in this study [4 vs. 14% lying within a 5 h period (25)]. The time difference in lying may have had minimal impact in terms of the welfare of the virtually-fenced animals as cattle will still rest while standing (10) but more comprehensive physiological assessments would be needed to confirm this. The FCM assessments did not show chronic stress in the virtually-fenced over electric tape animals. However, further replication and extension of the total trial time would confirm if this small difference in lying time equates to a significant welfare impact across months. The time budget differences seen between cohorts may have been related to weather conditions as the average temperatures were several degrees higher for cohort 1 and shade availability within all paddocks was limited (Figures 1, 7).

Overall, the FCM samples did not demonstrate that the cattle were experiencing different physiological stress responses to the fence types. The FCM patterns did however match the observed behavioral temperaments of the animals as groups/cohorts, behaviorally confirming our interpretation of the FCM results (29). The cattle were sourced off-site following commercial rearing and were not well-accustomed to handling when they arrived on site. Personnel worked with the cattle in the weeks before the trial commenced to reduce their adverse responses to handling and ensure the neckbands would be able to be fitted with relative ease. All groups showed the highest FCM responses at the end of their first week within the paddock (the baseline samples), and the concentrations steadily reduced across time with no observed peak following fence activations. Cohort 2 also showed significantly lower FCM concentrations overall which could have been a result of the increased time to become accustomed to the new housing environment, or the reduced temperatures experienced in comparison to cohort 1.

All cattle within the virtual fencing groups showed a reduction in the number of electrical stimuli received across time. However, as per previous studies with beef and dairy cattle using the same system, the individual variation in how quickly individual animals learned to respond to the audio cue, and how frequently they interacted with the fence was variable (6, 8, 30). There was no individual that was identified as being unable to learn within these groups which is a positive indication of the stimuli being controllable and predictable for the animals (9). Producers indicate there are always individual animals that respond poorly to physical electric fences (pers. comm. DLMC 2019) and it is possible that the same may occur for virtual fencing technology where some individuals continually break through the virtual boundary. This was not observed for the 32 animals tested in the current study, but this may still be a possibility within larger herds of cattle. Interestingly, there were clear differences between groups in how rapidly the animals learned to stay within the inclusion zone. There were also some animals that had a peak in received signals later in the trial. This could have been due to an increase in motivation to cross over into new pasture but more longer-term studies are needed to document how responses to a virtual line change across time to enable a greater understanding of the technology's application. Social facilitation may play a role in the learning process but the extent to which this occurs is currently poorly understood. The audio tone is designed to be heard only by the individual wearing that neckband, but calm weather conditions and close proximity may enable other animals to hear tones from herd mates' neckbands. A previous trial with an earlier prototype of the same system suggested an effect of social facilitation as there were avoidance responses to the audio cues by cattle that had not yet received an electrical pulse (8); this was also found in the current study. Cows observing herd mates receiving gentle handling were more likely to keep a shorter distance to the gentle handler following the observations of their neighbors (31). Cattle in rangeland will eat novel plants if following observation of consumption by herd mates (32). But the role of social facilitation and/or social dominance in the learning of virtual fencing cues is still to be quantified. It is also unclear how individual cattle temperament impacts on learning of a virtual fencing system. Individual cattle show different reactivity to their first experiences with an automated milking system (33) and animals with more reactive temperaments responded less to training in the milking parlor than less reactive individuals (34). No specific temperament assessments were carried out on the cattle in this study. This would be a valuable area for further research to determine whether temperament and/or cattle breed affects the reactions to the audio cues and electrical pulses and consequently learning ability and willingness to stay within the prescribed area.

Overall, this trial is the first longer-term assessment of the impacts of virtual fencing as compared to a widely-used form of physical electric fencing. There were minimal differences between the two fence groups within the 4-week time period in terms of paddock utilization, body weight, and FCM concentrations. Further trials should confirm these findings as well as look at longer-term impacts of small differences in lying time and the effects of animal temperament on individual and group learning variation. Trials over an even greater period of time with larger animal group sizes would be a valuable addition to the current knowledge to ensure cattle continue to stay within prescribed areas using virtual boundaries and to determine if there are individual cattle that may not be suited to this technology.

## Data Availability Statement

The datasets generated for this study will not be made publicly available. Some of the data (behavioral and fecal cortisol) could be provided upon request. But none of the data from the neckband devices due to being commercial in confidence.

## Ethics Statement

The animal study was reviewed and approved by the Chiswick Animal Ethics Committee, CSIRO.

## Author Contributions

DC, JL, and CL contributed conception and design of the study. DC, JL, and HK organized the database. DC and HK performed the statistical analyses. DC wrote the first draft of the manuscript. DC, JL, HK, and CL wrote sections of the manuscript. All authors contributed to manuscript revision, read, and approved the submitted version.

### Conflict of Interest

Agersens Pty Ltd provided technical support with the neckbands but did not contribute to the study design, data analysis, or the decision to publish the results. The authors declare that the research was conducted in the absence of any commercial or financial relationships that could be construed as a potential conflict of interest.
